# Small but Mighty: Ten Myths and Misunderstandings About the Cerebellum

**DOI:** 10.1162/nol_e_00152

**Published:** 2024-08-15

**Authors:** Julie A. Fiez, Catherine J. Stoodley

**Affiliations:** Departments of Psychology, Neuroscience, and Communication Science and Disorders, Learning Research and Development Center, Center for the Neural Basis of Cognition, University of Pittsburgh, Pittsburgh, PA, USA; Developing Brain Institute and Center for Prenatal, Neonatal and Maternal Health Research, Children’s National Hospital, Washington DC, USA; Departments of Neuroscience and Pediatrics, The George Washington School of Medicine and Health Sciences, Washington, DC, USA

This special issue of *Neurobiology of Language* focuses on the role of the cerebellum in spoken and written language comprehension and production. The volume brings together behavioral and neural evidence bearing upon this question using an array of methods. As editors, we are excited by the collective impact of this work, which includes recent findings from many of the leading researchers who study the cerebellum and language. We also find ourselves pondering the term “special” as a reflection of the widespread tendency of brain researchers to comfortably relegate the cerebellum to a minor role in cognition. As a result, our 21st century understanding of the cognitive neuroscience of the cerebellum is not yet consistently recognized by the field, leading to an underappreciation of the cerebellar contributions to language beyond its role in the coordination of articulation. Here we offer a “top ten” list aimed at countering some of the myths and misunderstandings that keep it out of the limelight.

## SMALL BUT MIGHTY

The name of the cerebellum—Latin for “little brain”—is a good starting point for considering ways in which the cerebellum is underestimated. It is true that the cerebellum is volumetrically smaller than the cerebrum. However, with over 50 billion neurons it has more than twice as many neurons as the cerebral cortex (von Bartheld et al., 2016). Given that neurons are often regarded as the basic computing unit of the brain, this fact alone leads us to ask, “How could a structure with more than half the human brain’s neurons not have achieved star status?” Further, the lateral cerebellar hemispheres have expanded along with association regions of the cerebral cortex (Herculano-Houzel, 2010), indicating that these two brain systems evolved together. Indeed, this was one of the factors that led Leiner et al. (1986) to posit a role for the cerebellum in cognitive functions. This comparative neurology perspective has been a sufficient argument to link, for example, the prefrontal cortex to human cognition; we suggest that the same should be true for the cerebellum.

## MY ENIGMA IS YOUR ENIGMA

Many of the activation maps of cognitive terms in the Neurosynth database (https://neurosynth.org/) contain cerebellar clusters with significance values rivalling those of cerebral cortical clusters. Unfortunately, cerebellar findings often receive little discussion or are ignored entirely beyond inclusion in a table reporting significant foci. As to why this is the case, we proffer our personal experience of hearing comments such as, “We see the cerebellum all the time, but we just don’t know what to say about it,” a theme of inscrutability that has been echoed by cerebellar researchers themselves. Open questions of course remain, but that is true for every brain region. We see no reason to believe that the cerebellum should be far less understood, or much harder to understand, than Broca’s area, Wernicke’s area, the visual word form area, the angular gyrus, anterior temporal pole, or other brain regions commonly associated with speech and language processing.

## TWO BRAINS IN ONE HEAD

One clear point is that the cerebellar and cerebral cortices have fundamentally distinct cytoarchitectures that undoubtedly endow them with differing computational capacities. The cytoarchitecture of the cerebellar cortex involves three (not six) layers, variations in its organization are revealed most readily by molecular markers rather than differences in neuronal composition, its principal output is inhibitory rather than excitatory and involves a neuron (the Purkinje cell) found only in the cerebellum, and the basic computational circuit of the cerebellum, the microzone, diverges in organization from the columns of the cerebral cortex (Apps & Hawkes, 2009). These variations in circuitry and neurotransmitter systems alter the neurovascular coupling that is the foundation for functional neuroimaging (Diedrichsen et al., 2010) and underlie differences in the basic representational structure of the cerebellum. For instance, the cerebellum contains multiple full body maps without a clear hierarchical representation to each other, and with a fractured somatotopy in which discrete patches can represent non-contiguous body parts, in contrast to the hierarchically and topographically organized sensory and motor homunculi in the cerebrum (Apps & Hawkes, 2009; Xue et al., 2021). Given such distinctions, an overarching consideration is that research assumptions based on the cerebral cortex may not successfully transfer to the cerebellum.

## OUT OF SIGHT, OUT OF MIND

The location and orientation of the cerebellum makes it easier to overlook and harder to study. It is slightly tucked under the cerebrum, extends about 25 mm below the cerebral ventral surface, and is less prone to stroke-related focal injury (Subramanian et al., 2009). Thus, across the major methods of modern cognitive neuroscience (neuroimaging, neurophysiology, lesion studies), the path of least resistance is to simply ignore the cerebellum. Functional magnetic resonance imaging (fMRI) studies frequently refer to a “whole brain” analysis, but many studies use acquisition parameters that do not provide full coverage of the cerebellum, make exclusionary analysis choices such as defining regions via atlases that do not include the cerebellum, or use software packages with defaults that mask out the cerebellum. Neurophysiological methods (electroencephalography [EEG], magnetoencephalography [MEG], and transcranial magnetic stimulation [TMS]) have faced different methodological obstacles related to the distance and orientation of the neuronal populations that give rise to the signals that propagate to the skull or that are affected by imposed electromagnetic fields (Andersen et al., 2020; van Dun et al., 2017). Neuropsychological studies have been hampered by challenges in identifying and recruiting participants with focal brain injury isolated to the cerebellum, especially the sample sizes needed for rigorous analysis of structure–function correspondences, and studies involving other etiologies of cerebellar dysfunction face similar issues. It is heartening that progress has been made; for example, advances in neuroimaging have increased the efficiency of acquiring brain volumes with full coverage of the cerebellum and provided higher-resolution cerebellar atlases (Diedrichsen et al., 2010), new analytic approaches have been developed for extraction of EEG and MEG signals (Samuelsson et al., 2020), new stimulation protocols have been developed for cerebellar TMS and related methods (Grimaldi et al., 2014), and patient research registries hold promise as a way to recruit larger samples of individuals with isolated cerebellar dysfunction (Traschütz et al., 2021). Collectively, these and future advances offer exciting new tools for shining the research spotlight squarely on the cerebellum.

## LOCATION, LOCATION, LOCATION

Even when the cerebellum is considered, an important source of misunderstanding is the tendency to treat it as a unitary structure with a single function. The patterning of cerebellar anatomical connectivity gives rise to functional subregions within the cerebellum, including regions that are clearly associated with vestibular, oculomotor, and sensorimotor control, which are the evolutionarily older cerebellar subregions. The newer lateral cerebellar hemispheres interconnect with major cerebral cortical networks to support a range of cognitive and affective processes. Task-related activity patterns and resting state functional connectivity networks identified within the cerebellum largely align and selectively interface with these major cerebral cortical networks, including those associated with speech and language processing (Seitzman et al., 2020; Xue et al., 2021). Location thus provides a vital context for the functional interpretation of neuroimaging, neurophysiological, and neuropsychological findings in the cerebellum, as it does in the cerebrum. In considering location, it is important to recognize that functional maps of the cerebellum align poorly with lobule boundaries (e.g., King et al., 2019) and so strategies based on neuroanatomy may be less suitable than in the cerebral cortex. Fortunately, the creation and continued advancement of cerebellar-specific atlases and precision within-participant mapping has been pivotal in moving forward knowledge and analytic tools for functional localization within the cerebellum.

## MOTOR, MOTOR, MOTOR—SURELY IT MUST BE MOTOR?

Damage to the cerebellum is characterized by loss of coordinated voluntary movement that leaves an individual more prone to stumbles and falls, dysarthric speech, and poor eye movement control. These impairments can be readily observed and were described as hallmarks of cerebellar damage more than 75 years ago (Holmes, 1939). The undoubted role of the cerebellum in motor control has been in part a boon, because it has driven very profitable theoretical and empirical advances that have been fruitfully extended to the study of its contributions to cognition and language. But it has also been in part a bane, by promoting a motor-centric double standard in which a higher bar is set for in the cerebellum as compared to the cerebrum for interpreting a finding as cognitive rather than motor, when in fact cerebellar regions tend to coactivate with their functionally connected cerebral counterparts (Seitzman et al., 2020; Tripathi & Somers, 2023). Consequently, the interpretational lens for cognitive measures is often cerebro-centric, even when there is no principled reason to reach a different interpretation for functionally coupled cerebro-cerebellar regions.

## HAVEN’T YOU HEARD ABOUT THE PERSON BORN WITHOUT A CEREBELLUM?

There is a “neuromyth” of case studies in the literature that document intact motor and cognitive functions in individuals with complete developmental agenesis of the cerebellar hemispheres. It is true that such cases can be identified, but in all well-studied cases there was evidence of motor impairments, such as delayed walking and dysarthric speech, and cognitive impairments, such as delayed language, descriptions of low intelligence, and poor educational outcomes (Glickstein, 1994; Leck & Pickett, 2022; Yu et al., 2015). Similar motor and cognitive delays and lasting impairments have been observed with high frequency in all other forms of developmental cerebellar macroscopic malformations, with more severe malformations generally associated with poorer motor and cognitive outcomes (Bolduc & Limperopoulos, 2009). Additionally, there is strong evidence linking two of the most common neurodevelopmental conditions, autism spectrum disorder and attention deficit-hyperactivity disorder, with changes in cerebellar anatomy, connectivity, and function (Stoodley, 2016). Finally, developmental cerebellar injury, most commonly in association with treatment of a medulloblastoma tumor or preterm birth, is frequently associated with persistent motor and cognitive impairments (Cantelmi et al., 2008; Dellatolas & Câmara-Costa, 2020; Tavano et al., 2007). Overall, the developmental literature provides solid and compelling evidence for a critical role of the cerebellum in motor and cognitive development.

## UNIMPAIRED DOES NOT MEAN UNAFFECTED

From an evolutionary perspective, it is difficult to rationalize the sheer expense (volume, energy consumption) of the human cerebellum if it does not provide a robust behavioral advantage. It may therefore be surprising that the evidence associating cerebellar dysfunction with specific cognitive impairments remains mixed, especially in adult populations. One likely factor is that cerebellar patients are often compared as a group to a neurotypical control population, an approach that often stems from the challenges in sample recruitment, but which disregards the location of injury or dysfunction as a critical variable (Stoodley et al., 2016). Another factor is that the cerebellum functions more as a critical supporting actor than the obvious star of the show. In the motor domain, cerebellar damage is not associated with paralysis, but rather the loss of well-coordinated, appropriately timed movement (Holmes, 1939); in the language domain, cerebellar damage is not associated with aphasia, but rather the loss of well-coordinated, appropriately timed speech and sentence processing (Mariën et al., 2014; Tavano et al., 2007; Urban, 2013). Affected individuals may “get by” using reserve cerebellar capacity and other neural systems together with compensatory strategies (Mitoma et al., 2020), achieving a level of ability that allows them to score within normal limits on many neuropsychological assessments (which, as a side note, have been largely designed from a theoretical framework of cerebral function and dysfunction). But this does not mean that individuals with cerebellar dysfunction are able to perform at the top of their game or easily acquire new information. As points of comparison, individuals with damage limited to anterior perisylvian cortex tend to recover from Broca’s aphasia and yet this region is still widely accepted as central to language (Fridriksson et al., 2015); language skills can be remarkably preserved following a left hemispherectomy at an early age (Ramantani & Reuner, 2018), and yet the lateralization of language to the left cerebral hemisphere continues to be a firmly held belief.

## FASTER, BETTER, SMARTER

Indeed, it can be argued that the cerebellum does not house the representations that underlie language, but instead acts upon that information to facilitate language processing, optimizing performance. When we think about what differentiates “good” cognition, it is often not just that a complex task can be performed at all, but that performance is fast, accurate, flexible, and responsive to the unexpected. In locomotion, we want the ability to navigate uneven terrain, quickly and accurately reach for our coffee, and perform basic tasks automatically. In language we want to use prediction to maintain the flow of conversation in degraded conditions (e.g., a noisy bar) and we want to be flexible enough to effortlessly comprehend sentences with syntactic structures and word choices that violate our expectancies. Given that the cerebral cortex and the cerebellum are interconnected in ways that allow bidirectional information exchange, a key challenge is to determine the relative roles of each system and how they influence each other (Kawato et al., 2021). In language, the information loop between right cerebellar lobule VII and the left inferior frontal gyrus is an example of a system that is commonly co-activated during semantic prediction, and new data emerging from methods with better temporal resolution (MEG; Alho et al., 2023) show high functional coherence between these regions during language processing. The effective connectivity between the cerebellum and cerebral cortex is modulated by task conditions, indicating a dynamic system that adapts depending on task demands. Theoretical considerations of this dynamic interplay propose that it reflects the use of cerebellar internal models to predict what is coming next, enabling rapid, automatic performance, and to process feedback signals, allowing high levels of performance to be sustained in the context of perturbations and deviations from predictions (Ito, 2008; Sokolov et al., 2017).

## LEARNING BY DOING

Humans of course are not born with the capacity to skillfully interact with the world. Our motor, language, and cognitive abilities develop and adapt based on our ongoing experiences throughout our lifespan. Given the vast learning capacity of the human brain, there is a need to consider learning mechanisms that go beyond forming and evaluating statistical co-occurrences, since otherwise a biologically implausible amount of training is needed to ensure the generalization of learned knowledge (i.e., avoid overfitting; Kawato et al., 2021). Recent advances in artificial intelligence illustrate this point, as the training of large language models requires exposure to more words than a human will experience in their lifetime. We proffer that it is time to give serious attention to the cerebellum and the power of its architecture to serve as the premier “learning machine” for the cerebral cortex. The cerebellum is distinguished by its role in supervised learning, with an architecture that in the motor domain is thought to support the learning and adaptation of two categories of internal models: forward models, which involve receiving copies of motor commands and predicting movement trajectories or sensory consequences, and inverse models, which involve receiving desired trajectories and computing the necessary motor commands to realize those trajectories. Extending these ideas to the cognitive domain provides the intriguing potential to explain a number of computationally and theoretically vexing problems, such as how large and complicated internal models can be learned with a small number of trials and without an external “teaching signal” (Kawato et al., 2021). Thus, as we move through our lives, the cerebellum may use feedback about performance outcomes and model prediction errors to seamlessly form, improve, and hierarchically connect internal models of action and cognition that capture and optimize our interactions with the world. This leads us to suggest that a greater focus on the role of the cerebellum in dynamic aspects of language, that is, learning and adaptation, will yield interesting insights regarding the role of the cerebellum in receptive and expressive language.

## GOING FORWARD …

Updated scanners allow for higher resolution scanning and more of the brain to be imaged in a short period of time. Cerebellar atlases are available for localization of findings and region-specific analyses. New analytical techniques for MEG/EEG enable the extraction of cerebellar signals. Our appreciation of cerebellar functional subregions is helping to clarify the sometimes-conflicting evidence from cerebellar clinical populations. More sophisticated neuromodulation approaches are available that allow us to test hypotheses about the specific contributions of the human cerebellum to language. And we have the computational capacity to deal with very large quantities of data. Many of the *methodological* challenges that initially hampered the study of the human cerebellum have been resolved, and theoretical models of the cerebellum and its cognitive capacities continue to evolve in response to empirical findings. Here, we hope that we have helped clarify some of the *conceptual* challenges that have limited the incorporation of the cerebellum into our understanding of the broader neural networks that support a range of cognitive processes—including the comprehension and production of language.

## FUNDING INFORMATION

Julie A. Fiez, National Institutes of Health (https://dx.doi.org/10.13039/100000002), Award ID: R01 HD096738. Julie A. Fiez, National Institutes of Health (https://dx.doi.org/10.13039/100000002), Award ID: R15MH126404. Julie A. Fiez, National Institutes of Health (https://dx.doi.org/10.13039/100000002), Award ID: R15CA271450. Catherine J. Stoodley, Department of Defense, Award ID: W81XWH-19-1-0249.
